# Unique Metabolic Contexts Sensitize Cancer Cells and Discriminate between Glycolytic Tumor Types

**DOI:** 10.3390/cancers15041158

**Published:** 2023-02-11

**Authors:** Jonathan A. Chacon-Barahona, Jeffrey P. MacKeigan, Nathan J. Lanning

**Affiliations:** 1 Department of Biological Sciences, California State University, Los Angeles, CA 90032, USA; 2 Pediatrics and Human Development, College of Human Medicine, Michigan State University, Grand Rapids, MI 49503, USA; 3 Department of Cell Biology, Van Andel Research Institute, Grand Rapids, MI 49503, USA

**Keywords:** cancer metabolism, glycolysis, oxidative phosphorylation, pentose phosphate pathway, mTOR signaling, glycolytic tumors

## Abstract

**Simple Summary:**

We sought to assess cancer cell viability in the context of glycolytic versus oxidative phosphorylation carbon source availability from cell lines and expression data from variably glycolytic human tumors. We performed an RNAi screen of genes consisting of the cytosolic machinery for ATP production and regulation of bioenergetic output in cancer cells in glycolytic or oxidative phosphorylation (OXPHOS) conditions. We identified the pentose phosphate pathway as requisite for viability under glycolytic conditions and mTOR signaling as requisite for viability under OXPHOS conditions. We then characterized gene sets within this panel to identify similarities and differences amongst RNA-seq profiles across variably glycolytic cancer types. This analysis identified glycolytic tumor profiles from non-glycolytic tumor profiles. Our analyses support classification of tumors by metabolic phenotype.

**Abstract:**

Cancer cells utilize variable metabolic programs in order to maintain homeostasis in response to environmental challenges. To interrogate cancer cell reliance on glycolytic programs under different nutrient availabilities, we analyzed a gene panel containing all glycolytic genes as well as pathways associated with glycolysis. Using this gene panel, we analyzed the impact of an siRNA library on cellular viability in cells containing only glucose or only pyruvate as the major bioenergetic nutrient source. From these panels, we aimed to identify genes that elicited conserved and glycolysis-dependent changes in cellular bioenergetics across glycolysis-promoting and OXPHOS-promoting conditions. To further characterize gene sets within this panel and identify similarities and differences amongst glycolytic tumor RNA-seq profiles across a pan-cancer cohort, we then used unsupervised statistical classification of RNA-seq profiles for glycolytic cancers and non-glycolytic cancer types. Here, Kidney renal clear cell carcinoma (KIRC); Head and Neck squamous cell carcinoma (HNSC); and Lung squamous cell carcinoma (LUSC) defined the glycolytic cancer group, while Prostate adenocarcinoma (PRAD), Thyroid carcinoma (THCA), and Thymoma (THYM) defined the non-glycolytic cancer group. These groups were defined based on glycolysis scoring from previous studies, where KIRC, HNSC, and LUSC had the highest glycolysis scores, meanwhile, PRAD, THCA, and THYM had the lowest. Collectively, these results aimed to identify multi-omic profiles across cancer types with demonstrated variably glycolytic rates. Our analyses provide further support for strategies aiming to classify tumors by metabolic phenotypes in order to therapeutically target tumor-specific vulnerabilities.

## 1. Introduction

Cancer cells undergo metabolic adaptations in order to meet biosynthetic requirements and address homeostatic challenges [1,2]. Advances in understanding cancer metabolism have relied on data from individual cell lines, animal models, and patient samples. The past 15 years of research on this front have revealed diverse mechanisms of cancer metabolism, from specific metabolic alterations promoting oncogenesis [3,4] to metabolic diversity and plasticity driving particular cancer phenotypes [5,6]. When assessing results investigating metabolic changes, it is now clear that nutrient availability can significantly impact biological outcomes both in vitro and in vivo [7,8]. Therefore, additional assessment of nutrient-specific effects on cancer cells as well as delineation of gene sets in cancers that rely on different metabolic strategies may provide an additional perspective to this field. 

To address this, we chose to focus on glycolytic versus non-glycolytic metabolic programs. It is well known that many cancers demonstrate enhanced glycolysis, a phenotype that is often associated with tumor aggressiveness and malignancy [9,10,11]. Many reasons for enhanced glycolysis have been identified, such as the necessity for glycolytic intermediates to supply biosynthetic pathways or the production of reducing equivalents [1]. However, the causes and consequences of this metabolic shift in cancer still remain incompletely understood. Some propose that increased aerobic glycolysis allows cancer cells to sustain large pools of glycolytic intermediates which, in turn, promotes pathways branching off of glycolysis, such as the pentose phosphate pathway–an essential step underlying nucleotide biosynthesis [12,13]. Others propose that increased flux of glucose into glycolysis enhances the overall synthesis of ATP and other molecules needed to support proliferation, given the relative kinetic differences in ATP production [14,15,16] and potential to generate reducing equivalents between OXPHOS and glycolysis [17,18,19]. While each of these proposals has considerable merit, none of them are without their drawbacks and caveats [19].

Equally interesting is the observation that while some cancer types have a high glycolytic rate, others rely less on glycolysis for energy production. The use of glycolysis-associated transcriptomic gene signatures and ^18^F-fluorodeoxyglucose positron emission tomography measurements allows for glycolysis intensity to be measured across different tumor types [20,21,22]. While the factors giving rise to differences in glycolytic rate amongst cancers remain incompletely understood, perturbed expression in glycolysis driver genes seems to play an important role [20], which itself is associated with genetic copy number alterations (CNAs) in glycolysis genes within tumors [23]. 

Here, we analyzed all glycolytic genes previously shown to characterize cancer-specific glycolysis levels [20] and expand on this signature to include genes in additional pathways associated with glycolysis under different nutrient availabilities to interrogate cancer cell reliance on glycolytic programs. Using an siRNA library targeting this gene panel, we analyzed cell viability in cells provided with either only glucose or only pyruvate as the major bioenergetic nutrient source. This analysis revealed the pentose phosphate pathway (PPP) as requisite for glycolytic-dependent viability and mTOR signaling as requisite for OXPHOS-dependent viability. We extended our analysis by characterizing similarities and differences in RNA-seq profiles across a pan-cancer cohort of variably glycolytic cancers—cancers defined based on glycolysis scoring as reported by [20]—using this gene set. In this context, KIRC, HNSC, and LUSC had the highest glycolysis scores, meanwhile, PRAD, THCA, and THYM had the lowest. Our analysis identified additional multi-omic profiles across variably glycolytic cancer types.

## 2. Materials and Methods

### 2.1. Cells and Reagents

HeLa (cervical carcinoma), U251 (glioblastoma), SF295 (glioblastoma), SF539 (glioblastoma), and SNB-75 (glioblastoma) cells were maintained in media formulated from DMEM lacking glucose, glutamine, and pyruvate (Sigma-Aldrich, St. Louis, MO, USA), and supplemented with 10 mM glucose or 10 mM pyruvate as indicated. All media was supplemented with 10% fetal bovine serum (FBS). Cells were grown in normal tissue culture-treated flasks under standard growth conditions of 5% CO_2_. Iodoacetic acid, rotenone, oligomycin, and 6-aminonicotanimide were obtained from Sigma-Aldrich, and AZD8055 was obtained from Selleck Chemicals, Boston, MA. All small interfering RNAs (siRNAs) were from Qiagen and were transfected into cells with oligofectamine (Invitrogen). Genes targeted and Gene IDs are provided in Supplementary Table S1. Knockdown levels were determined using quantitative reverse transcription-PCR (qRT-PCR) as previously described [24]. 

### 2.2. Viability, Cell Number, and ATP/Cell Measurements

All viability assays were performed in 96-well black-walled, clear bottom plates. Growth media was removed following indicated treatments, and 100 µL of a room-temperature solution of Cell Titer-Glo and Opti-MEM was immediately added to each well (1:1 Cell Titer-Glo:Opti-MEM). Luminescence (Cell Titer-Glo) readings were taken after 10 min room-temperature incubation. Student’s *t*-test was used to determine the significance of specified gene knockdown values from non-targeting siRNA values. All cell number assays were CyQUANT measurements, and were performed in 96-well black-walled, clear bottom plates. Growth media was removed following indicated treatments and cells were processed according to the manufacturer’s protocol. Student’s *t*-test was used to determine the significance of specified gene knockdown values from non-targeting siRNA values. All ATP/cell assays were performed in 96-well black-walled, clear bottom plates. Growth media was removed following indicated treatments, and 100 µL of a room-temperature solution of Cell Titer-Glo, Opti-MEM, and CyQUANT was immediately added to each well (1:1:0.0058 Cell Titer-Glo:Opti-MEM:CyQUANT). Luminescence (Cell Titer-Glo) and fluorescence (CyQUANT) readings were consecutively taken after 10 min room-temperature incubation. Cell Titer-Glo values were divided by CyQUANT values to generate ATP/cell values. Inhibitors and drug treatments: Cells were treated with iodoacetic acid (IAA, 10 μM), oligomycin (500 ηM), or DMSO (vehicle, equivalent percent as drug) for four hours prior to ATP/cell measurements. Cells were treated with AZD8055 at the indicated concentrations for 18 h prior to cell viability measurements. Cells were treated with 6-aminonicotinamide (6-AN, 100 μM) or DMSO (equivalent percent as drug) for 24 h prior to cell number measurements.

### 2.3. RNAi Screen

HeLa cells were plated at a density of 500 cells per well in black-walled, clear bottom 384-well plates in DMEM containing 25 mM glucose, 1 mM pyruvate, and 4 mM glutamine, and 10% FBS. Eight hours after cells were plated, the media was switched to DMEM containing 10% FBS and either 10 mM glucose or 10 mM pyruvate. Sixteen hours after the media change, cells were transfected in each nutrient condition in duplicate with a pool of 2 siRNAs per gene (25 nM per siRNA) and 2 µL/mL HiPerfect. A total of 24 and 48 h after siRNA transfection, fresh media was added to the cells. Seventy-two hours after siRNA transfection, growth media was removed, and 30 µL of a room-temperature solution of Cell Titer-Glo/Opti-MEM was immediately added to each well (1:1 Cell Titer-Glo:Opti-MEM). Luminescence was taken after a 10 min room-temperature incubation. 

Eight wells of each plate were transfected with cell death control siRNAs (AllStars Hs Cell Death siRNA) for transfection quality control. Eight additional wells were transfected with non-targeting siRNAs (AllStars Negative Control siRNA) for plate normalization. Pooled siRNA for each gene was transfected in each nutrient condition in duplicate, with duplicate transfections occurring in separate plates. Luminescence values for each well were normalized to the average value of non-targeting siRNAs for each respective nutrient condition in each plate. 

### 2.4. Hierarchical Clustering

To determine the optimal number of clusters for the viability data sets, K-means clustering was performed to assemble groups of genes that when knocked down elicited similar effects on viability. Here, the base R kmeans( ) function was used to compute dissimilarity amongst different groups of genes using the Euclidean Distance metric [25,26]. This method was applied to scaled and centered siRNA panel data. To determine the optimal number of clusters, the fviz_nbclust( ) function of the factoextra package v1.0.7 was used. The use of this function allowed for visual determination of optimal cluster amount via the elbow and silhouette method [27,28]. Having determined the optimal cluster amount, the viability data set was then grouped via hierarchical clustering to also assess the impact of media restriction on observed changes in viability.

### 2.5. cBioPortal Data Query and Processing

Pan-cancer copy number alteration and batch-normalized mRNA expression data for the gene panel were queried using cBioPortal [29]. These data represent genomic and transcriptomic alterations in primary tumors of glycolytic cancers KIRC, HNSC, and LUSC, and relatively non-glycolytic cancers PRAD, THCA, and THYM—relative to peritumoral control tissues. Since mRNA expression values for ENO4 were missing across all samples, this gene was removed from all further analyses. 

### 2.6. Sparse Principal Component Analysis

To take an unsupervised statistical learning approach to assess which genes from the gene panel were the most influential in forming tumor-specific clusters, a sparse principal component analysis (sPCA) was applied to KIRC, HNSC, LUSC, PRAD, THCA, and THYM tumor mRNA expression profiles were used for the gene panel using the mixOmics package v6.14.0 [30,31]. mRNA expression profiles were centered and scaled prior to parameter tuning using the base R scale( ) function. sPCA components and variable optimization were determined using the mixOmics’ tune.spca( ) function, which uses repeated M-fold cross-validation of partitioned training and testing sets. Parameter tuning was done using 5-folds, selecting the optimal number of variables per component after 50 repeats.

### 2.7. Copy Number Alteration Analysis

To assess genomic alterations across genes that cluster variably glycolytic cancer transcript profiles, genes that were selected as loadings for the optimized sPCA components (PC1-3) were used as inputs for copy number alteration (CNA) analysis. Copy-number levels are derived from the Genomic Identification of Significant Targets in Cancer (GISTIC) algorithm, which sets copy-number levels at −2 (Deep Deletion; likely homozygous deletion), −1 (Shallow Deletion; likely heterozygous deletion), 0 (Diploid; no loss or gain in copy-number), +1 (Gain; few broad copy-number gains), and +2 (Amplification; many focal copy-number gains) [32,33]. CNA data were queried for KIRC, HNSC, LUSC, PRAD, THCA, and THYM primary tumors using cBioPortal [29], and CNA profiles with at least one alteration were visualized using OncoPrints [34]. 

### 2.8. Pathway Enrichment Analysis

To assess the underlying biological pathways in genes that cluster variably glycolytic cancer transcript profiles, genes that were selected as loadings for the optimized sPCA components (PC1-3) were subjected to enrichment analysis using ShinyGO, which tests input gene sets for enrichment against a robust range of gene sets from Gene Ontology, KEGG, UniProt, WikiPathways, and other molecular pathway databases [35]. For the PC1-3 loadings enrichment analyses, all available pathway databases were considered for enrichment, and the top 20 pathways were selected at an FDR cutoff of <0.05.

## 3. Results

### 3.1. siRNA Screen in Glycolytic versus OXPHOS Conditions

**Figure 1:**
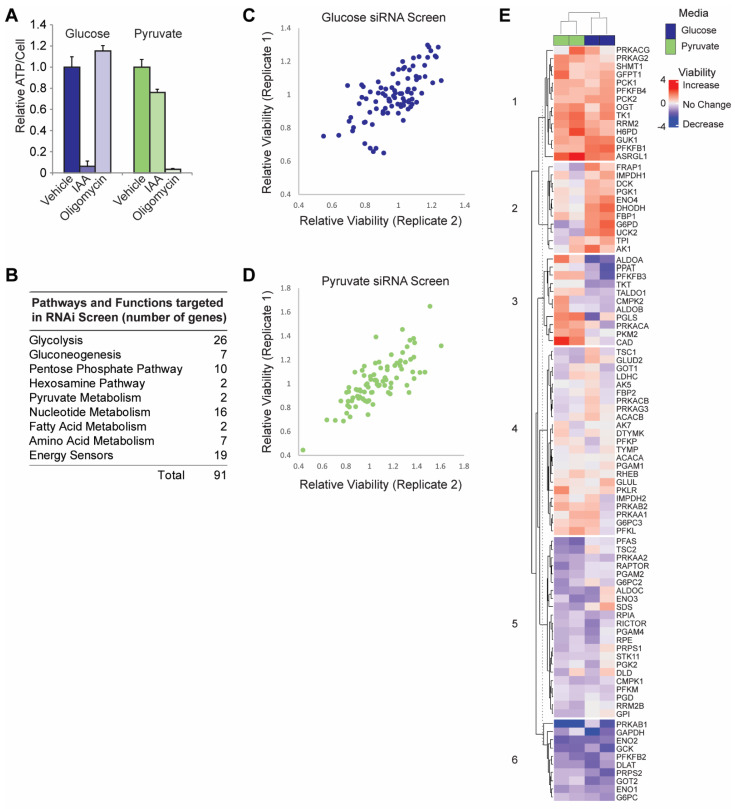
Metabolic-sensitized RNAi screen. (**A**) HeLa cells cultured in glucose or pyruvate were treated with a glycolytic inhibitor (IAA, 10 μM) or an OXPHOS inhibitor (oligomycin, 500 nM) for four hours, and ATP/cell was assessed to verify unique media conditions restricting cells to either glycolytic or OXPHOS bioenergetics. (**B**) Gene categories in siRNA screen. (**C**) Relative viabilities of cells in glucose media following siRNA knockdown. (**D**) Relative viabilities of cells in pyruvate media following siRNA knockdown. (**E**) Hierarchical clustering of siRNA screen results.

To initially assess the contribution of glycolytic and metabolic regulator genes to bioenergetic output and cellular homeostasis, we chose HeLa cells as a model cancer cell line that is highly glycolytic, but which also possesses the flexibility to adapt its metabolic programs to changing nutrient source availability [8,36,37,38]. We maintained HeLa cells in media formulated with specified carbon bioenergetic sources, a strategy we and others have previously shown to effectively restrict cultured cells to specific metabolic programs [8,39]. To validate these conditions, cells in each condition were treated with either a glycolytic inhibitor (IAA) or an OXPHOS inhibitor (oligomycin), and relative ATP/cell was measured [8]. Figure 1A demonstrates that cells cultured with glucose as the exclusive bioenergetic carbon source are especially sensitive to IAA while cells cultured with pyruvate as the exclusive bioenergetic carbon source are especially sensitive to oligomycin. Cell cultured under these conditions were subjected to an siRNA screen targeting genes encoding energy-sensing signaling proteins as well as all glycolytic enzymes and enzymes in pathways associated with glycolysis, (Figure 1B, Table S1) and cell viability was assessed (Figure 1C,D).

Hierarchical clustering of the screen results yielded clustering changes on the basis of carbon source (Figure 1E), further underscoring the ability of the nutrient source to force a specific bioenergetic program. Cluster 6 genes are heavily represented by glycolysis genes whose knockdown robustly decreased viability regardless of carbon source. By contrast, cluster 1 consisted of genes whose knockdown robustly increased viability across both media conditions and is largely represented by pathways that shuttle central carbon metabolism away from ATP synthesis. Both hexosamine genes, both rate-limiting genes for gluconeogenesis, two ATP-consuming glycolysis genes, and the entry point into the pentose phosphate pathway are in this cluster. Clusters 2 and 3 reveal differential effects of gene knockdown dependent on nutrient source/bioenergetic program. Knockdown of cluster 3 genes generally increased viability under OXPHOS conditions while decreasing viability under glycolytic conditions. This cluster contains PKM2 which is the pyruvate kinase isoform most highly associated with the oncogenic phenotype [40,41], the two PPP genes required for carbon reentry into glycolysis, as well as two aldolase genes that are required for glyceraldehyde 3-phosphate (G3P) production. G3P is also the metabolite produced by carbons reentering glycolysis from the PPP. Cluster 2 represents the opposite effect with viability increasing under glycolytic conditions and dropping under OXPHOS conditions. This cluster contains the rate-limiting enzyme for the PPP as well as many nucleotide metabolism genes. Throughout the screen, gene isoforms as well as members of the same metabolic pathway behaved differently under the same metabolic conditions. For example, suppressing PFKFB1 and PFKFB4 expression increases viability across both conditions while PFKFB2 suppression decreases viability across both conditions and PFKFB3 suppression selectively reduces viability in glucose. Similarly, suppression of AMPK subunit isoforms produces comparable variable effects. Variable effects have been the hallmark of the past two decades of cancer research on metabolic enzyme isoforms and pathways [42,43,44,45,46,47], as underscored by our siRNA screen.

### 3.2. Validation of RNAi Screen Hits

**Figure 2:**
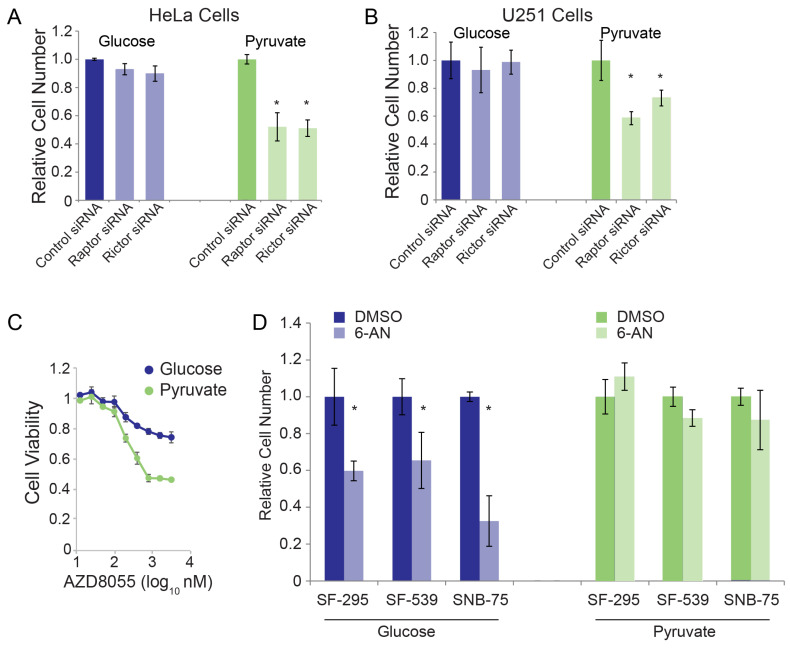
Sensitization of cells under restricted nutrient sources. (**A**) HeLa and (**B**) U251 cells were cultured in glucose or pyruvate-restricted medias and transfected with the indicated siRNAs. Seventy-two hours post transfection, cell numbers were assessed. (**C**) HeLa cells cultured in glucose or pyruvate-restricted media were treated with the indicated doses of mTOR inhibitor AZD8055. (**D**) Glioblastoma cell lines cultured in glucose or pyruvate-restricted medias were treated with the PPP inhibitor, 6-AN, followed by assessing cell numbers. Asterisks indicate *p* < 0.05 by Student’s *t*-test.

Our screen results suggest perturbation of mTOR signaling selectively reduces viability under OXPHOS conditions while the perturbation of the PPP selectively reduces viability under glycolytic conditions (Figure 1E and Figure S1). We confirmed that silencing mTOR signaling components (rictor or raptor) selectively reduced HeLa cell viability in OXPHOS conditions (Figure 2A), and these results were also consistent in glioblastoma cells (Figure 2B). Chemical inhibition of mTOR using the catalytic small molecule inhibitor AZD8055 produced similar results, reducing viability in OXPHOS conditions to a greater extent than in glycolytic conditions (Figure 2C). Chemical inhibition of the PPP selectively inhibited viability in glycolytic conditions in three different glioblastoma cell lines, in line with our siRNA screen results in HeLa cells (Figure 2D). These results are consistent with our siRNA screen, and further highlight selective sensitivities when cells are forced into specific metabolic programs. 

### 3.3. Identifying Altered Transcript & Genetic Profiles in Cancers with High or Low Glycolytic Profiles

The siRNA screen results provide a useful tool for contextualizing results related to metabolic genes, pathways, and programs in cultured cells. However, we sought to extend our analysis into biospecimens from cancer patients. We chose two sets of cancer types for this analysis—one set of glycolytic cancers and one set of non-glycolytic cancers defined on the basis of glycolysis scoring, as previously reported [48]. Kidney renal clear cell carcinoma (KIRC), Head and Neck squamous cell carcinoma (HNSC), and Lung squamous cell carcinoma (LUSC) defined as the glycolytic cancer group while Prostate adenocarcinoma (PRAD), Thyroid carcinoma (THCA), and Thymoma (THYM) defined the non-glycolytic cancer group. To identify sets of altered gene expression profiles for our gene panel in these glycolytic and non-glycolytic cancer types, an unsupervised statistical classification analysis via sPCA was applied to cancer patient primary tumor RNA-seq profiles from TCGA for highly glycolytic cancers HNSC (*n* = 523), KIRC (*n* = 512), and LUSC (*n* = 487), and relatively less glycolytic cancers PRAD (*n* = 494), THCA (*n* = 500), and THYM (*n* = 123). A full description of the clinical demographics for queried cancer cohorts may be found in Table S2. Preliminary clustering outlined nine patient tumor transcript profiles as outliers which were removed from further analysis (Figure S2A,B). Variable selection on each principal component (PC) revealed that the optimal number of genes per component was 17 genes for PC1, 11 genes for PC2, and 28 genes for PC3 (Figure S2C).

**Figure 3:**
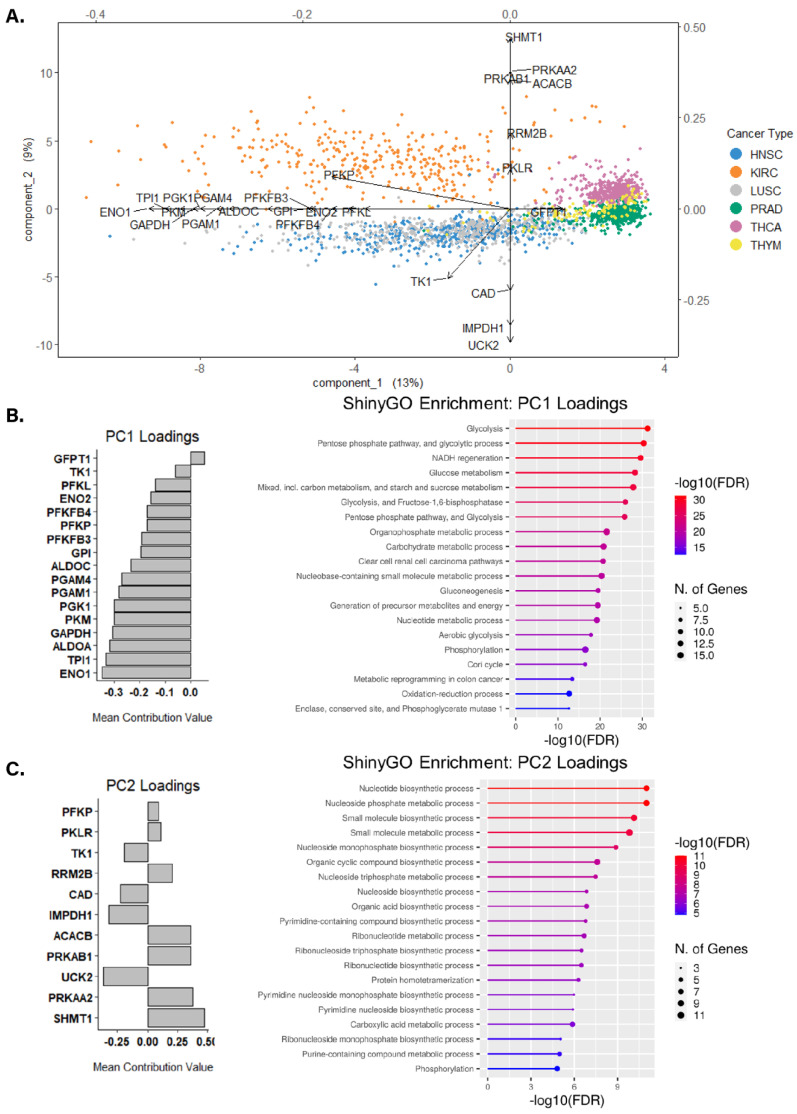
(**A**) sPCA of pan-cancer gene set transcript profiles. Biplot of unsupervised clustering for two components of the final sPCA result along with PC1-2 loadings. (**B**,**C**) ShinyGO enrichment of PC1-2 loading gene sets. (**B**) PC1 loadings comprise almost entirely of genes involved in glycolysis. PC1 loadings contribute to the enrichment of glycolysis, cellular redox, and metabolic reprogramming pathways, in addition to diverse biosynthetic pathways. (**C**) PC2 loadings comprise of genes involved in nucleotide metabolism and AMPK signaling, with the most significantly enriched pathways relating to nucleotide metabolism.

sPCA feature selection was evaluated via a correlation circle plot, which plots the correlation of each gene’s transcript expression along axes made up of the first two PCs. Compared to the first two PCs in the initial untuned correlation circle plot, the variable-selected sPCA result generated a set of variables that were highly correlated with the first two PCs, indicated by all selected genes organizing within the outer and inner limits of the unit circle (Figure S3A). Along PC1, the resulting unsupervised sPCA result clustered the tumor transcript profiles of HNSC, KIRC, and LUSC together and clustered PRAD, THCA, and THYM together (Figure 3A). Additionally, PRAD, THCA, and THYM tumor transcript profiles clustered together towards the center of PC2, whereas HNSC and LUSC profiles clustered together below the PC1 projection, and KIRC clustered above PC1 projection, showing distinct clustering of non-glycolytic tumor profiles–albeit to a lesser extent than was observed along PC1. Differences in clustering between high and low tumor glycolysis groups appeared less clear along PC3, though the most variable transcript profiles in this space were of KIRC, HNSC, THCA, and THYM cancers, with THCA, THYM, and HNSC showing the most similar profiles (Figure S3B). 

**Figure 4:**
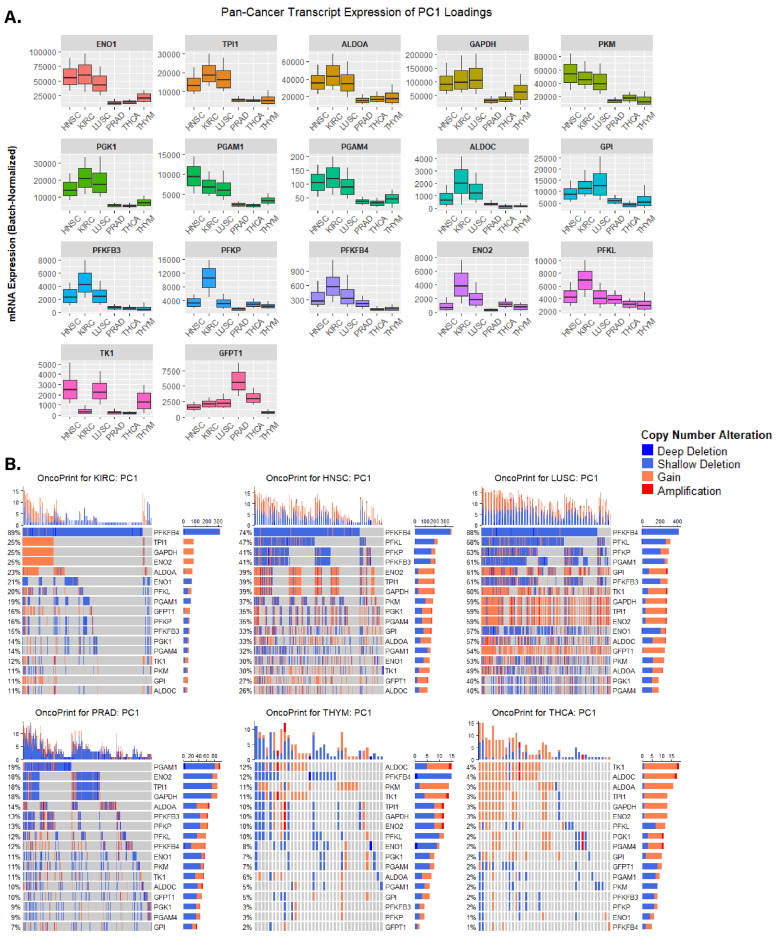
mRNA and CNA profiles for PC1 loadings across the variably glycolytic pan-cancer cohort. (**A**) Boxplots showing transcript expression distributions of sPCA’s PC1 loadings across cancer cohorts. Due to excessive variability in bulk mRNA expression across all samples, the top and bottom 10th percentiles were excluded here. (**B**) Oncoprints of CNAs in PC1 loadings across the cancer cohorts.

To preliminarily characterize transcript and genetic differences in the sPCA-selected PC loadings between glycolytic and non-glycolytic cancer types, batch-normalized mRNA expression and CNAs of genes within each PC loading were visualized across our pan-cancer cohort. For PC1, the 17 genes were primarily involved in glycolysis, apart from TK1 (nucleotide metabolism) and GFPT1 (hexosamine pathway)–both of which had the lowest contribution to the PC1 loadings (Figure 3B,C). Generally, expression of PC1 genes involved in glycolysis was higher in glycolytic tumors compared to less glycolytic tumors, with differences in the expression being most prominent in ENO1, TPI1, ALODA, GAPDH, PKM, PGK1, and PGAM1/4, which expectedly aligned with their highly ranked contributions to PC1 loadings (Figure 4A). When assessing CNA profiles for PC1 loadings in our pan-cancer cohort, glycolytic cancer types had more overall CNAs compared to CNA abundance in non-glycolytic cancers (Figure 4B). Furthermore, the most frequently altered PC1 genes, PFKFB4, had substantial copy-number loss via shallow deletions across glycolytic cancers. This widespread loss was not observed in non-glycolytic cancer types. Interestingly, while PFKFB4 had a widespread copy-number loss in glycolytic cancer types, its transcript expression was markedly higher in glycolytic cancers compared to non-glycolytic cancers.

PC2’s 11 genes were collectively involved in numerous pathways including serine glycine/nucleotide/fatty acid metabolism, AMPK signaling, and glycolysis (Figure 3C). When looking at glycolytic cancers, bulk mRNA expression of PC2 genes in KIRC differed from that of HNSC and LUSC, explaining the difference in clustering between KIRC and HNSC/LUSC (Figure S4A and Figure 3A). For many PC2 genes, bulk mRNA expression was either markedly high or low in the HNSC/LUSC cluster, and the opposite in KIRC, but relatively moderate in non-glycolytic tumor types. The expression of PKLR was unexpectedly low for all non-KIRC samples. Similar to observations reported for PC1’s CNA profiles, CNA profiles for PC2 reveal trends within and between glycolytic and non-glycolytic cancer types (Figure S4B). Generally, glycolytic tumor samples had higher CNAs overall, apart from PRAD, which had a similar CNA abundance to KIRC. HNSC and LUSC had the highest CNA prevalence amongst PC2 genes, in particular for RRM2B, where ~70% of evaluated samples in either cohort had copy number gains for this gene. RRM2B CNAs were not as abundant in KIRC, but were the highest alteration in PRAD tumor samples, with 33% of evaluated PRAD samples having a gain or amplification in RRM2B copy number. Interestingly, while THCA genetic profiles revealed that only 2% of evaluated patient tumor samples had CNAs in RRM2B, bulk mRNA expression of RRM2B was highest in THCA tumor samples.

While PC3 in the final sPCA result did not organize clusters on the basis of low or high glycolysis groupings, at the extreme ends of each, there was still some discernible overlap in clustering for HNSC, THCA, and THYM (Figure S3B). It is important to note, however, that most tumor transcript profiles clustered similarly along PC3. The 28 genes for PC3 were collectively associated with a diverse range of pathways, akin to the makeup of PC2 loadings, with additional genes involved in mTOR signaling, gluconeogenesis, glutamine metabolism, and pentose phosphate pathway (Figure S5A). As expected, the PC3 transcript profile did not show remarkable differences in mRNA expression between glycolytic and non-glycolytic tumors. Expression of PRKAA1 (AMPK signaling), which held the highest contribution amongst PC3 loadings, was elevated in KIRC, LUSC, and PRAD tumor samples, while only being moderately or lowly expressed in HNSC, THCA, and THYM tumor samples. Interestingly, many transcript profiles for PC3 genes revealed distinctive biomarkers of mRNA expression within this pan-cancer cohort. For example, AK1 expression was high only in THCA, and GFPT1 was only elevated in LUSC. Similarly, the expression of ACACA and CPMK1 was only elevated in PRAD, and the expression of TSC2 and RRM2 was only increased in THYM. Finally, the expression of TYMP was only elevated in HNSC. It is important to note that these cancer-specific transcriptional biomarkers were not particularly unique at the genetic copy number level, apart from extensive copy number gains for GFPT1 in LUSC, and overall CNAs for TYMP in HNSC (Figure S5B). Additionally, widespread copy number gains for PRKAA1 and RICTOR were found in glycolytic cancers, and extensive shallow deletions for DLAT were found in HNSC and LUSC. Widespread shallow deletions for PFAS were also found in LUSC and PRAD, and copy number gains for PRKAG2 were found across the entire pan-cancer cohort.

### 3.4. Assessing Biological Themes in Genes Clustering Variably Glycolytic Cancers

To evaluate extended biological pathways associated with genes within each PC loading set, each PC loading set was subjected to ShinyGO enrichment analysis (Figure 3B,C). As expected, the most significantly enriched term for PC1 gene set enrichment was “Glycolysis” (FDR < 0.05). Additionally, significant enrichment for the pentose phosphate pathway, NADH regeneration, aerobic glycolysis, and metabolic reprogramming was also detected (Figure 3B), indicating the PC1 gene set comprises genes that collectively influence the degree of aerobic glycolysis in glycolytic cancer types. Enrichment analysis of the PC2 gene set revealed the most significantly enriched pathways to be largely involved in nucleotide/nucleoside metabolism (Figure 3C). This highlights a metabolic axis that may underlie metabolic differences between glycolytic and non-glycolytic cancer types, given that the PC2 gene set clustered non-glycolytic tumor profiles apart from glycolytic tumor profiles (Figure 3A). While the PC3 gene set contained genes involved in numerous pathways, enrichment analysis of PC3 genes revealed the most significantly enriched pathways to be involved in AMPK/mTOR signaling and nucleotide metabolism (Figure S3C).

## 4. Discussion

Metabolic alterations in cancer cells remain incompletely understood, yet therapeutically promising drug targets. The gaps in knowledge surrounding oncogenic metabolic shifts are further complicated by the complexity of gene networks both directly involved with—or indirectly emanating from—glycolysis and glycolysis-associated pathways. In this study, we analyzed an siRNA panel across OXPHOS-promoting and glycolysis-promoting metabolic contexts. A broad assessment of bulk changes in cellular bioenergetics across all siRNA panels revealed that most knockdowns elicited media-dependent effects on viability (Figure 1). Hierarchical clustering of these panels identified clusters of knockdowns that elicited conserved and media-dependent changes in viability, with media-dependent changes mostly occurring under OXPHOS-promoting and glycolysis-promoting metabolic contexts (Figure 1). From these results, we verified individual susceptibilities of cancer cells in different metabolic contexts. Cells relying on glycolysis for bioenergetics are especially sensitive to PPP inhibition while cells relying on OXPHOS for bioenergetics are especially sensitive to mTOR pathway inhibition (Figure 2).

Unsupervised clustering via sPCA of the gene set using tumor RNA-seq profiles across glycolytic and non-glycolytic cancers effectively clustered glycolytic tumor profiles against non-glycolytic tumor profiles along PC1-2 (Figure 3). By looking at trends in expression across all tumor profiles for PC1-2 genes, and in looking at pathway enrichment for these two gene sets, it is apparent that the greatest sources of variation between tumor profiles of glycolytic and non-glycolytic cancer types come from the expression of glycolysis genes–which is expected given that the feature-selected PC1 gene set almost entirely recapitulates the glycolysis activity gene signature previously reported here by Wei et al. [20]. Interestingly, the second greatest source of variation between glycolytic and non-glycolytic tumor profiles was explained by genes involved in nucleotide metabolism. While variation in expression for glycolysis genes between glycolytic and non-glycolytic cancer types is expected, it was surprising to find that differences in expression for genes involved in nucleotide metabolism were able to cluster non-glycolytic tumor profiles together. This finding suggests that perturbed nucleotide metabolism may underlie the metabolic shift towards increased aerobic glycolysis in highly glycolytic cancers. It is important to note that glycolytic and non-glycolytic tumor profiles did not form unique clusters along PC3 (Figure S3B). By this logic, given that PC3 genes were enriched in AMPK and mTOR signaling pathways (Figure S3C), it is possible that perturbed AMPK and mTOR signaling do not strongly underlie metabolic shifts towards increased aerobic glycolysis in highly glycolytic cancers.

CNA profiles in glycolytic and non-glycolytic cancers also revealed unique CNAs in glycolytic cancers. Along PC1, PFKFB4, PFKL, and PFKP (glycolysis) genes had widespread deletions in the genomes of KIRC, HNSC, and LUSC patient tumors (Figure 4). Similarly, RRM2B (nucleotide biosynthesis), PRKAA1 (AMPK signaling), and RICTOR (mTOR signaling) had widespread copy number gains exclusively in glycolytic cancers, apart from RRM2B, which had copy number gains in ~30% of evaluated PRAD patient tumor genes (Figure 4, Figures S3 and S4).

## 5. Conclusions

Our RNAi screen highlights the differential effects of perturbing metabolic pathways when cells are in different metabolic environments and demonstrates that cancer cell vulnerabilities can be effectively attained in these contexts. Other groups have demonstrated similar results in vivo with other metabolic pathways [7]. Additional in vitro and in vivo studies cataloging similar effects will shed further light on the potential for cancer cell therapeutic sensitization through metabolic interventions. While these informatics results highlight unique transcriptional and genetic differences between glycolytic and non-glycolytic cancer types, they are not without their limitations. The sPCA, while effective at clustering glycolytic and non-glycolytic tumor profiles on the basis of genes involved in glycolysis and nucleotide metabolism, collectively explained ~30% of the variation in all tumor RNA-seq profiles, meaning that most of the variation in the tumor RNA-seq profiles remained unexplained in the final sPCA result. Future work could explore additional non-linear dimensionality reduction techniques, such as t-distributed stochastic neighbor embedding (t-SNE) or uniform manifold approximation and projection (UMAP)–both of which are widely used to organize transcriptome-level clusters [48,49]. In doing so, the deeper hierarchical structure of metabolic axes underlying glycolytic shift in cancer may be far better understood.

## Data Availability

For the informatics analyses, no new data were used in the present study. The results here are in whole or part based upon data generated by the TCGA Research Network: https://www.cancer.gov/tcga, and queried using cBioPortal: https://www.cbioportal.org/ (accessed on 20 January 2022). Unless otherwise specified, all analyses were performed using RStudio v1.3.959.

## Supplementary Materials

The following supporting information can be downloaded at: https://www.mdpi.com/article/10.3390/cancers15041158/s1, Figure S1: siRNA Screen Nutrient Comparison; Figure S2: Outlier filtering and variable selection for sPCA performed on gene set RNA-seq data; Figure S3: sPCA of pan-cancer gene set transcript proles; Figure S4: mRNA and CNA profiles for PC2 loadings across the variably glycolytic pan-cancer cohort; Figure S5: mRNA and CNA profiles for PC3 loadings across the variably glycolytic pan-cancer cohort; Table S1: Gene annotations Gene IDs for siRNA screen; Table S2: Pan-cancer patient demographics for gene set sPCA.
