# Supplementary material for: Unique Metabolic Contexts Sensitize Cancer Cells and Discriminate between Glycolytic Tumor Types
Source: Cancers (Basel). 2023 Feb 11;15(4):1158. doi: 10.3390/cancers15041158 (PMC9953999; doi:10.3390/cancers15041158)
Supplement: Supplementary file 1 [file cancers-15-01158-s001.zip › cancers-2188536-supplementary.pdf]

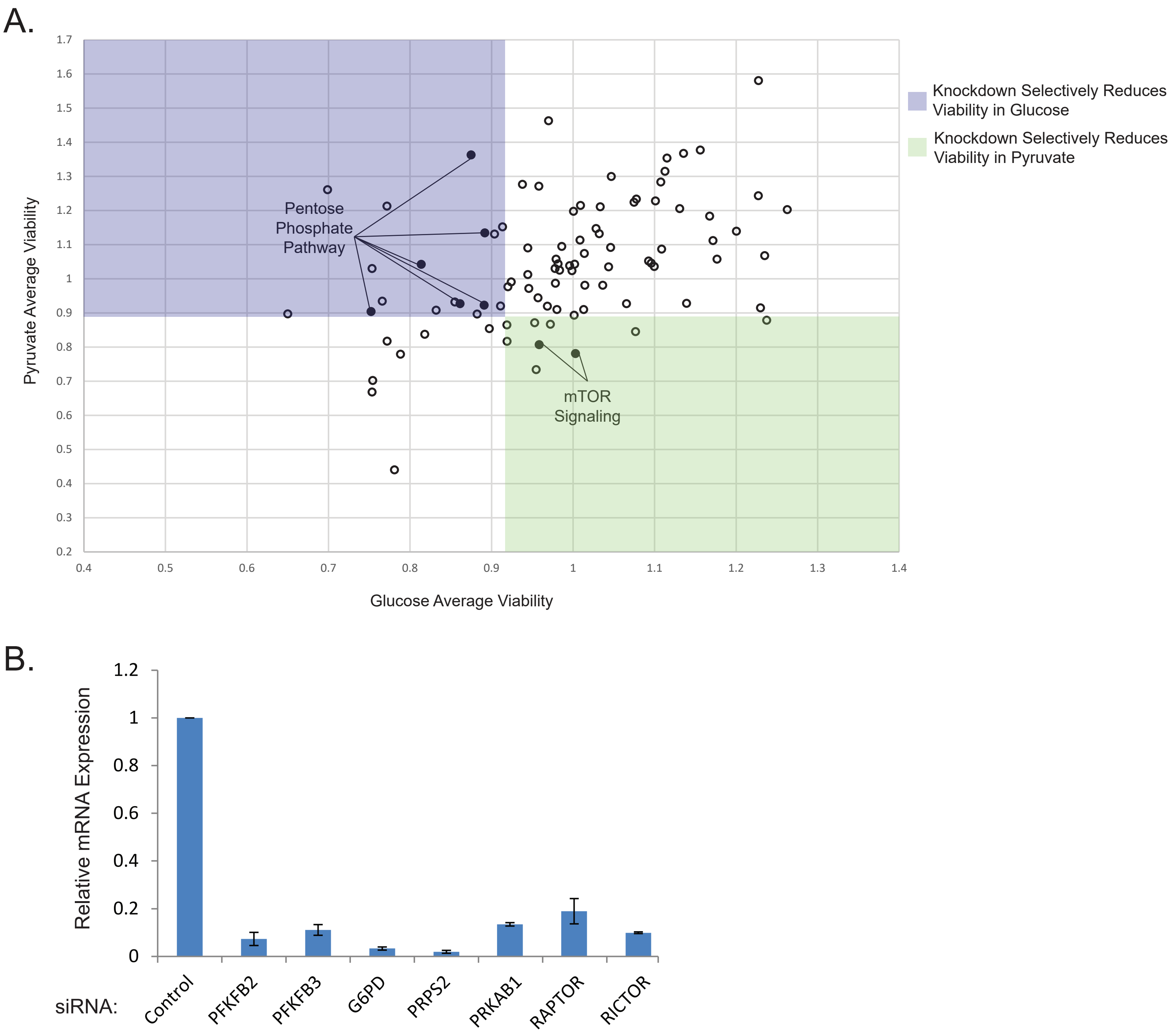

Figure S1. A. Comparision of differential nutrients in siRNA screen. Average viability from duplicate glucose screens (y-axis) were plotted against the average viability from duplicate pyruvate screens (x-axis). Genes whose knockdown selectively reduced viability three standard deviations from the control siRNAs are highlighted in blue (glucose screen) or green (pyruvate screen).
B. Knockdown analysis of selected siRNAs (two siRNA duplexes) from the siRNA screen. Total RNA was isolated from cells and primers were designed to specifically amplify the indicated genes.

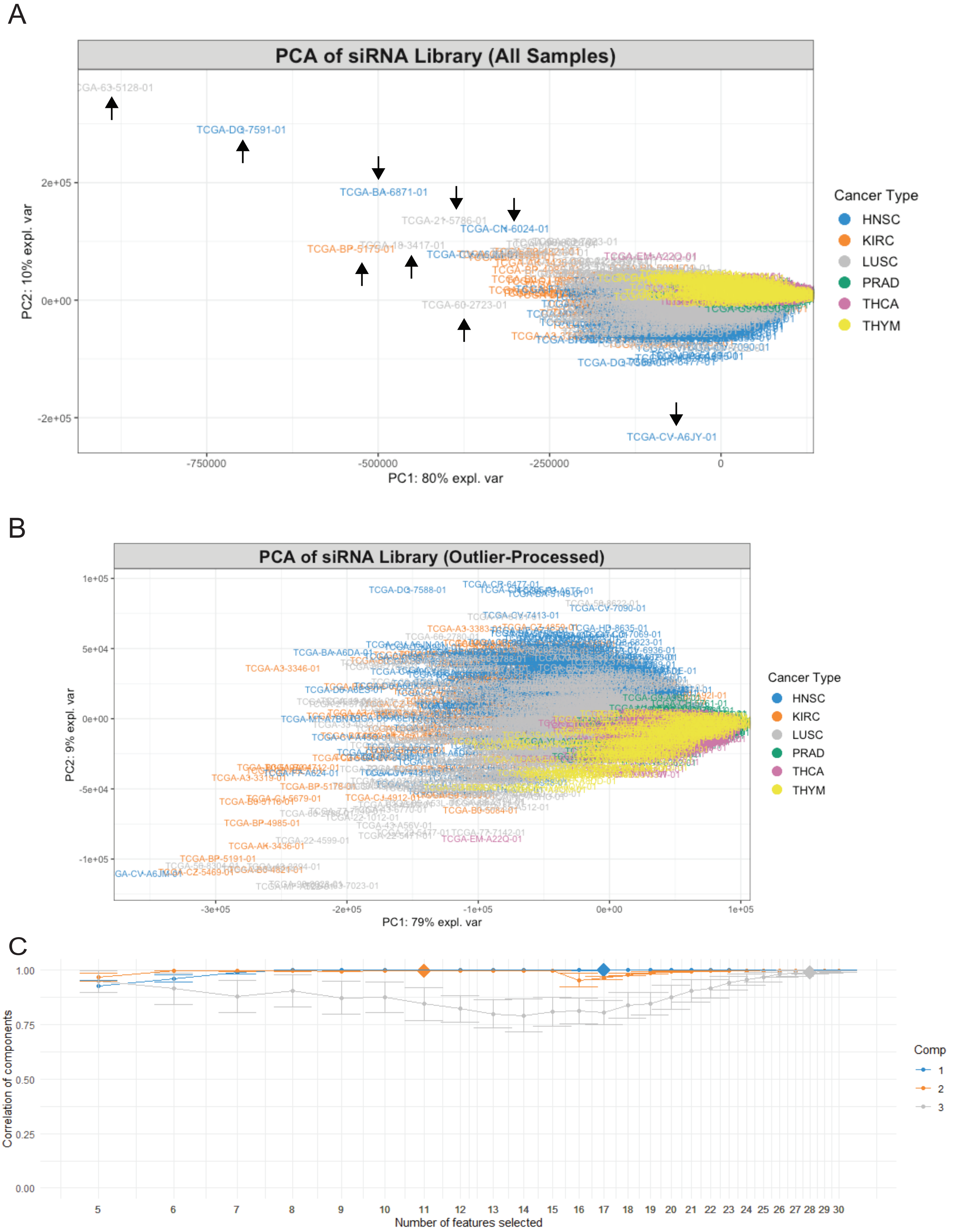

Figure S2. Outlier filtering and variable selection for sPCA performed on gene set RNA-seq data obtained from glycolytic (HNSC, KIRC, and LUSC) and relatively non-glycolytic (PRAD, THCA, and THYM) tumor samples. (A) Nine tumor samples were considered as outliers upon visual inspection and removed from further analysis. (B) sPCA after outlier removal. (C) Variable selection for the first three principal components.

A.

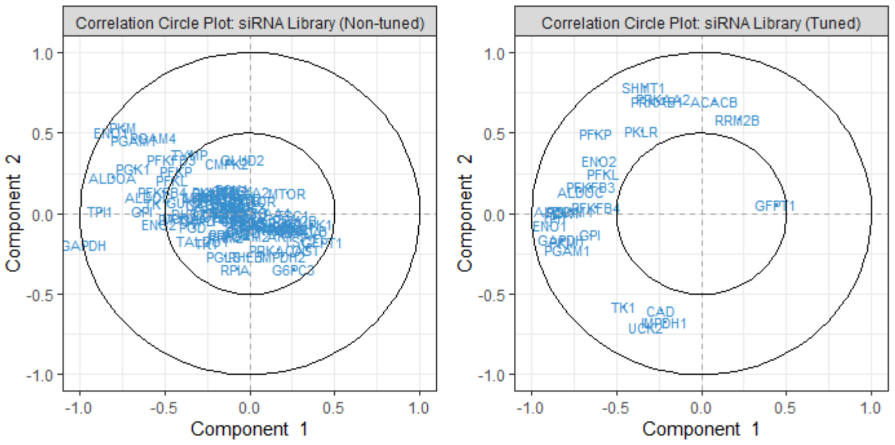

B.

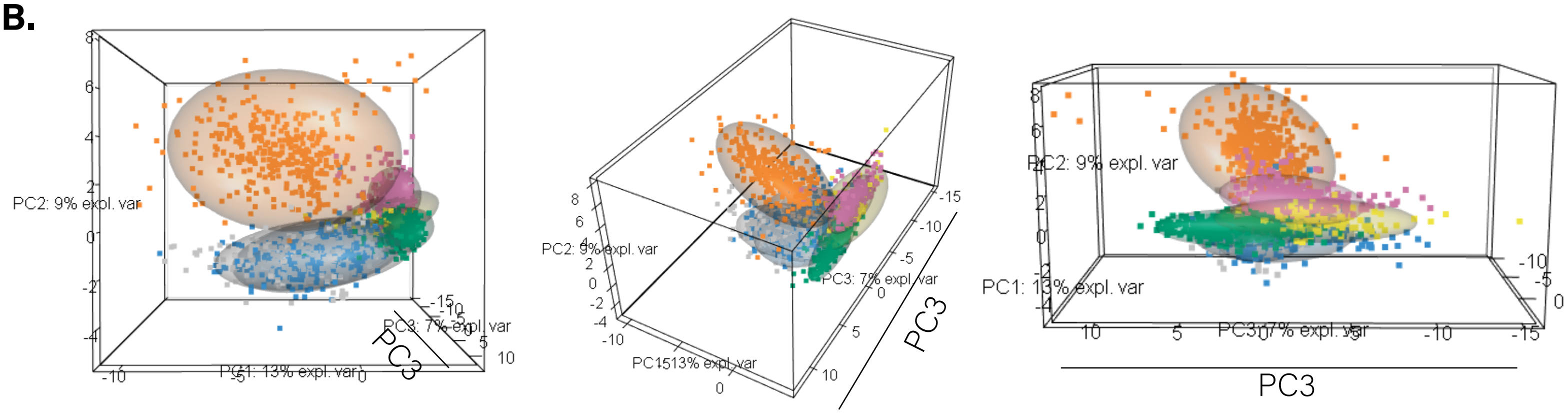

C.

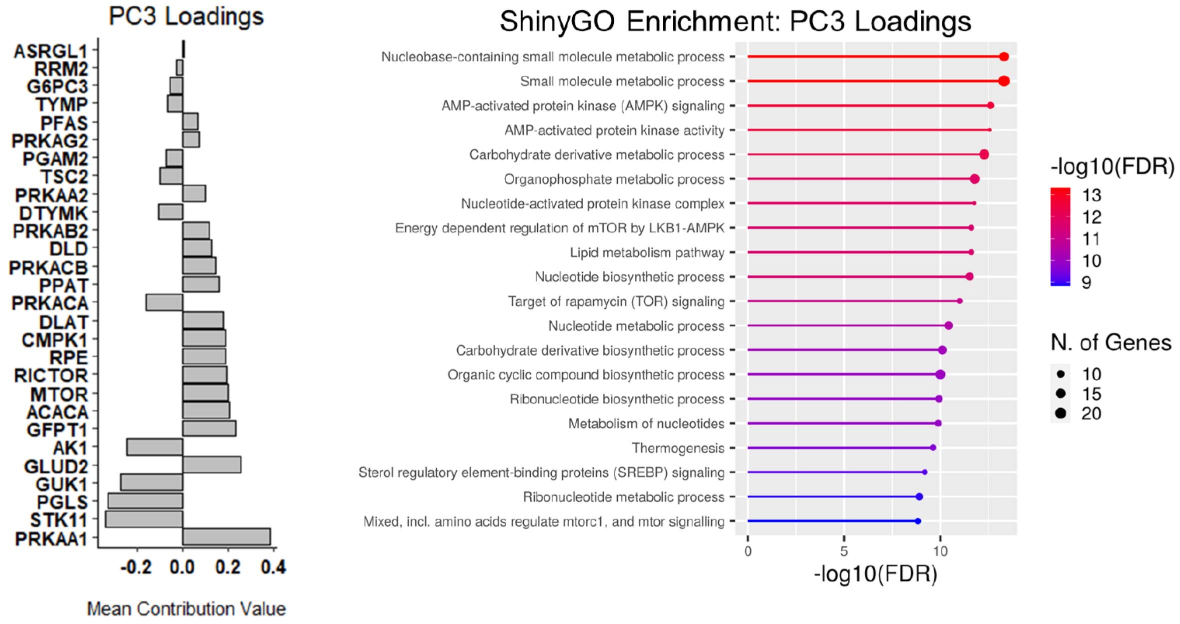

Figure S3. sPCA of pan-cancer gene set transcript profiles. (A) Correlation circle plot showing correlation, as standardized covariance, of each variable to a set of the first two principal components before and after variable selection. (B) Final 3D sPCA result showing unsupervised clus-tering in front, top, and side views along PC3. (C) PC3 loadings share a similar profile to that of PC2, and additionally contribute to enrichment of pathways related to AMPK/mTORC1 signaling.

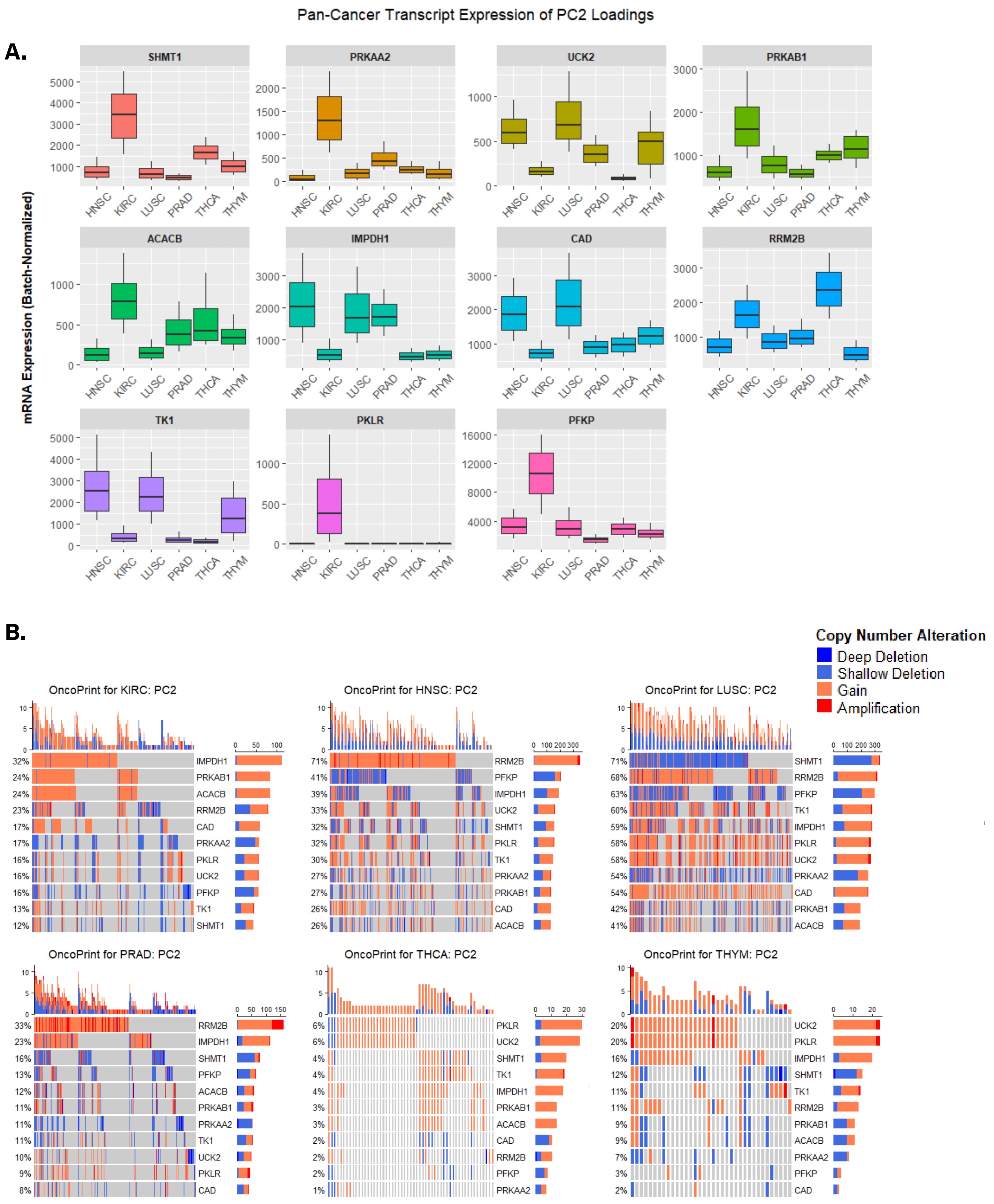

Figure S4. mRNA and CNA profiles for PC2 loadings across the variably glycolytic pan-cancer cohort. (A) Boxplots showing transcript expression distribution of sPCA's PC2 loadings across cancer cohorts. Due to excessive variability in bulk mRNA expression across all samples, the top and bottom 10th percentiles were excluded here. (B) Oncoprints of CNAs in PC2 loadings across the cancer cohorts.

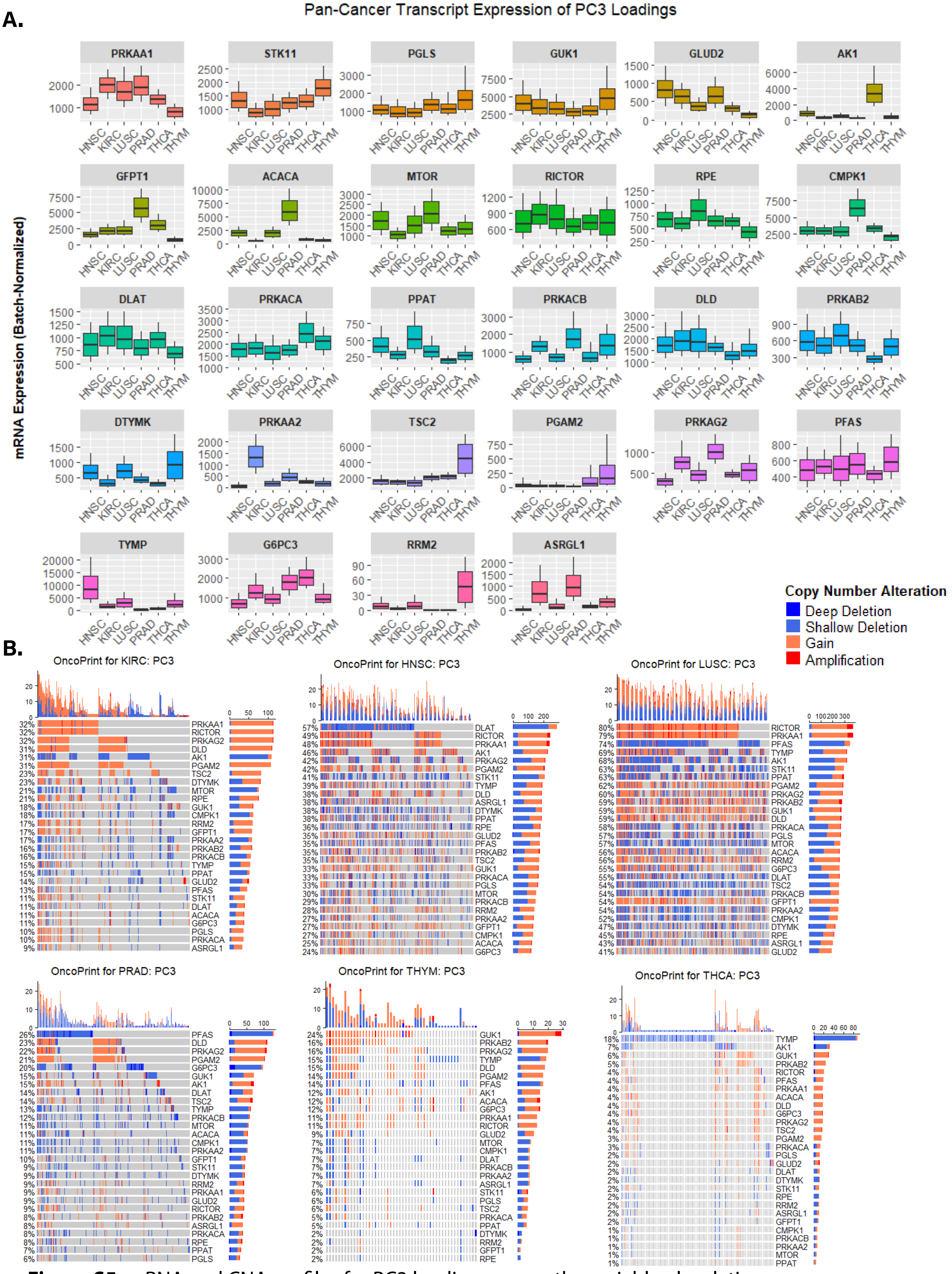

Figure S5. mRNA and CNA profiles for PC3 loadings across the variably glycolytic pan-cancer cohort. (A) Boxplots showing transcript expression distribution of sPCA's PC3 loadings across cancer cohorts. Due to excessive variability in bulk mRNA expression across all samples, the top and bottom 10th percentiles were excluded here. (B) Oncoprints of CNAs in PC3 loadings across the cancer cohorts.

Table S1. List of Genes Targeted in siRNA Screen

Entrez Gene Id	NCBI gene symbol	Entrez Gene Id	NCBI gene symbol	Entrez Gene Id
31	ACACA	3615	IMPDH2	253260
32	ACACB	3948	LDHC	6120
203	AK1	8473	OGT	22934
26289	AK5	5105	PCK1	6241
122481	AK7	5106	PCK2	50484
226	ALDOA	5198	PFAS	10993
229	ALDOB	5207	PFKFB1	6470
230	ALDOC	5208	PFKFB2	6794
80150	ASRGL1	5209	PFKFB3	6888
790	CAD	5210	PFKFB4	7083
129607	CMPK2	5211	PFKL	7086
1633	DCK	5213	PFKM	7167
1723	DHODH	5214	PFKP	7248
1737	DLAT	5223	PGAM1	7249
1738	DLD	5224	PGAM2	1890
1841	DTYMK	441531	PGAM4	7371
2023	ENO1	5226	PGD	
2026	ENO2	5230	PGK1	
2027	ENO3	5232	PGK2	
387712	ENO4	25796	PGLS	
2203	FBP1	5313	PKLR	
7889	FBP2	5315	PKM2	
2475	FRAP1	5471	PPAT	
2538	G6PC	5562	PRKAA1	
57818	G6PC2	5563	PRKAA2	
92579	G6PC3	5564	PRKAB1	
2539	G6PD	5565	PRKAB2	
2597	GAPDH	5566	PRKACA	
2645	GCK	5567	PRKACB	
2673	GFPT1	5568	PRKACG	
2747	GLUD2	51422	PRKAG2	
2752	GLUL	51422	PRKAG2	
2805	GOT1	53632	PRKAG3	
2806	GOT2	5631	PRPS1	
2821	GPI	5634	PRPS2	
2987	GUK1	57521	RPTOR	
9563	H6PD	6009	RHEB	

NCBI gene symbol
RICTOR
RPE
RPIA
RRM2
RRM2B
SDS
SHMT1
STK11
TALDO1
TK1
TKT
TPI1
TSC1
TSC2
TYMP
UCK2

Table S2. Pan-cancer patient demographics for gene set sPCA.

					**Race Category**		
Cancer	Samples	Male	Female	White	Black	Asian	NA/Other
KIRC	512	326	186	442	55	8	7
HNSC	523	382	141	448	47	11	17
LUSC	487	358	127	337	29	9	112
PRAD	494	494	0	147	7	2	338
THCA	500	134	365	328	27	52	92
THYM	123	64	59	102	6	13	2
